# Phase I study of chlorogenic acid injection for recurrent high-grade glioma with long-term follow-up

**DOI:** 10.20892/j.issn.2095-3941.2022.0762

**Published:** 2023-06-22

**Authors:** Zhuang Kang, Shan Li, Xun Kang, Jing Deng, Huarong Yang, Feng Chen, Jiandong Jiang, Jie Zhang, Wenbin Li

**Affiliations:** 1 Department of Neuro-oncology, Cancer Center, Beijing Tiantan Hospital, Capital Medical University, Beijing 100070, China; 2 Department of Oncology, Beijing Shijitan Hospital, Capital Medical University, Beijing 100038, China; 3 Sichuan Jiuzhang Biological Science and Technology Co. Ltd, Chengdu 610095, China; 4 Institute of Materia Medica, Chinese Academy of Medical Sciences and Peking Union Medical College, Beijing 100070, China

**Keywords:** Recurrent high-grade glioma, chlorogenic acid (CGA), temozolomide (TMZ)

## Abstract

**Objective:**

This study was aimed at analyzing the efficacy and safety of an injectable form of chlorogenic acid (CGA) in patients with recurrent high-grade glioma after standard of care treatments, through a first-in-human, open-label, dose-escalation phase I trial.

**Methods:**

A total of 26 eligible patients were enrolled, received intramuscular CGA injections at 5 dose levels, and were followed up for 5 years. CGA was well tolerated, and the maximum tolerated dose was 5.5 mg/kg.

**Results:**

The most common treatment-related adverse events occurred at the sites of injection. No grade 3 or 4 adverse events (e.g., drug allergy) were reported for these patients except for induration at the injection sites. A clinical pharmacokinetic study showed that CGA was rapidly eliminated from the plasma, with a t_1/2_ of 0.95–1.27 h on day 1 and 1.19–1.39 h on day 30, and no detectable CGA was observed on days 9, 11, 13, 23, 25, 27, and 29 before CGA administration. After the first treatment cycle, 52.2% of patients (12 of 23) achieved stable disease. Long-term follow-up indicated an estimated median overall survival of 11.3 months for all 23 evaluable patients. Of the 18 patients with grade 3 glioma, the median overall survival was 9.5 months. Two patients remained alive at the cutoff day.

**Conclusions:**

This phase I study demonstrated that CGA has a favorable safety profile (with no severe toxicity), and provides preliminary clinical benefits for patients with high grade glioma relapsing after prior standard therapies, thus shedding light on the potential clinical application of CGA for recurrent grade 4 glioma.

## Introduction

Glioma is a common and highly aggressive primary brain tumor in adults. Patients with grade 4 glioma have a mean overall survival (OS) of 9.7 months after primary treatment^1–3^. The standard care for patients with newly diagnosed glioblastoma is surgery followed by temozolomide (TMZ) administered concurrently with, and as an adjuvant to, radiation therapy. However, the disease recurs in almost all patients after the initial treatment, and the mean OS of patients with recurrent high-grade glioma (grade 4) is only 30 weeks. Currently, therapeutic options for patients with glioma are limited, and no standard therapy is available for patients with recurrent high-grade glioma. Multiple trials in recent years have evaluated the efficacy of nitrosoureas (e.g., lomustine), bevacizumab (a VEGF antibody), and other agents either alone or in combination with other treatments for recurrent grade 4 glioma, but the results of these trials have often been inconclusive, and the products tested have been associated with high toxicity and only minimal increases in OS^4^. Effective new therapeutic agents are thus urgently needed to meet this unmet medical need.

Chlorogenic acid (CGA) is a naturally occurring phenolic compound in many edible plants and fruits (e.g., eggplants and peaches), and is well known for its antioxidant and antibacterial activities as well as its ability to regulate lipids and glucose metabolism^5^. In recent studies, CGA has been demonstrated to have various anti-cancer activities, such as inhibition of oncogenic Bcr-Abl tyrosine kinase in leukemia cells, and inactivation of the activity of NF-κB and AP-1—promoters of tumorigenesis^6–9^. Furthermore, CGA enhances anti-cancer immunity and repolarizes macrophage from the anti-inflammatory M2 to the pro-inflammatory M1 phenotype^8^. More importantly, CGA has been found to penetrate the blood-brain barrier^10^ and significantly inhibits the growth of G422 glioblastoma in an orthotropic xenograft model^8^. These promising preclinical results provided the rationale for a phase I clinical trial investigating CGA in recurrent glioma. Here, we report the results of an open-label, dose-escalation phase I study aimed at evaluating the safety and tolerability of CGA, and preliminarily exploring CGA’s pharmacokinetic features and anti-tumor activity in patients with recurrent high-grade glioma.

## Materials and methods

### Study design and participants

This was an open-label, dose-escalation, phase I study. The primary objectives were to assess the safety and tolerability of CGA injection in patients with recurrent high-grade glioma. The secondary objectives were to characterize the pharmacokinetic profile and evaluate the antitumor activity of CGA injection. The primary endpoints were the maximum tolerated dose (MTD) and dose-limiting toxicity (DLT). The secondary endpoints were quality of life; the disease control rate, defined as the percentage of assessable patients with complete response, partial response, or stable disease (SD); and the OS, defined as the time from treatment allocation to death due to any cause. Changes in immune and cytokine markers were also assessed as exploratory endpoints. The study is registered in China (http://www.chinadrugtrials.org.cn, No. CTR20160113) and the USA (http://clinicaltrials.gov, No. NCT02728349).

Patients aged 18 years or older with histologically diagnosed advanced and recurrent glioma, including grade 3 and 4 glioma, for whom standard chemotherapy or radiotherapy failed, were eligible for this study. Eligible patients had a life expectancy longer than 3 months and a KPS score above 40. Additional eligibility criteria included white blood cell count ≥ 3.0 × 10^9^/L, neutrophil count ≥ 1.5 × 10^9^/L, platelet count ≥ 100 × 10^9^/L, plasma hemoglobin concentration ≥ 90 g/L, creatinine concentration≤ 1.5 times the upper limit of normal (ULN), aspartate and alanine aminotransferase concentrations ≤ 2.5 times the ULN, and total bilirubin ≤ 1.5 times the ULN. According to the Response Assessment in Neuro-Oncology Working Groupcriteria^11^, patients with the following symptoms or conditions were eligible for inclusion in the study, (1) consistent or increasing hormone therapy, (2) greater than 25% increases in enhancing lesions, and (3) changes in non-enhancement lesions confirmed to be caused by tumor progression, although these lesions cannot be measured. Patients who were pregnant, or who had a medical history of cardiovascular diseases, immunodeficiency disorders, diabetes, or nervous system diseases, were excluded. Patients with active viral infections (including hepatitis B, hepatitis C, syphilis, HIV infection, or AIDS-related illness) were also excluded. Patients who previously had irradiation administered to more than 30% of the bone marrow were not eligible. Patients who received surgery or radiotherapy within 4 weeks, nitrosoureas or mitomycin within 6 weeks, or tyrosine-kinase inhibitors within 2 weeks were also excluded. Moreover, patients who had used adrenal steroid hormones within 1 week and who required long-term treatments with adrenal steroid hormones were excluded. However, patients receiving corticosteroid nasal sprays, inhaled corticosteroids, and/or topical steroids were eligible.

The use of specimens and patient information was approved by the Ethics Committees of the Beijing Tiantan Hospital of Capital Medical University (Approval No. 2016004). All patients provided written consent for the use of their specimens and disease information for the study, in accordance with the Ethics Committee approval and the Declaration of Helsinki.

### Treatment protocol

This study was conducted in accordance with the Good Clinical Practice guidelines, the Declaration of Helsinki, and the corresponding regulations issued by the National Medical Products Administration. The protocol was approved by the institutional review board of Beijing Shijitan Hospital. Written informed consent was obtained from all participating patients, in accordance with the principles described by the National Medical Products Administration. CGA injection was administered intramuscularly to patients once daily for 28 days. Before the first dose, the patients received an intracutaneous drug allergy test. The initial dose was 2 mg/kg, and the dose was escalated to 7 mg/kg according to a conventional 3 + 3 design, and a modified Fibonacci method. Dose reductions were not allowed, but patients with DLT were permitted to restart treatment if the toxicity decreased to grade 1 or 2, and the previous treatment was paused within 1 week. After the first cycle of the 28-day treatment, patients were eligible to continue multiple-cycle treatment until investigator-determined disease progression, unacceptable adverse events, withdrawal of consent, or death.

### Pharmacokinetic studies

According to the preclinical animal data, the time points for the collection of single-dose and multiple-dose PK blood samples were as follows: (1) day 1, before administration, and 10, 20, 30, 45, 60, 90, 120, 150, 180, 240, 300, and 360 minutes after administration; (2) before administration in the morning on days 9, 11, 13, 23, 25, 27, and 29; and (3) day 30, before administration and 10, 20, 30, 45, 60, 90, 120, 150, 180, 240, 300, and 360 minutes after administration, to collect 3 mL of venous blood. Blood samples were centrifuged for 10 min at a speed of 3,000 r/min at 4 °C, and the plasma supernatant was transferred to a cryogenic tube for testing. The plasma concentration of CGA was measured with the LC-MS-MS method, and the main PK parameters, AUC_(0-t)_, AUC _(0-∞)_, t_1/2_, T_max_, Vz/F, CLz/F, and C_max_ were calculated. The pharmacokinetic parameters of CGA were calculated with the WinNonlin 6.5 linear compartment model and non-compartment model.

### Assessment

The National Cancer Institute Common Terminology Criteria for Adverse Events (Version 4.0) were used to grade adverse events. Blood samples were collected after single and multiple doses of CGA in all dose cohorts. Antitumor activities were assessed in all enrolled patients who received the first 28 days of treatment of CGA and for whom at least one post-treatment datapoint (brain MRI image) was available. Objective response and OS were summarized with descriptive statistics. The antitumor effects in patients were evaluated according to the Response Assessment in Neuro-Oncology Working Group criteria. The quality-of-life assessments were based on the European Quality of Life 5-Dimensions (EQ-5D) and Functional Assessment of Cancer Therapy (FACT-Br, version 4) questionnaires.

Safety data were collected for all patients who received at least one dose of CGA injection during the first 28 days of treatment (full analysis set). All adverse events occurring during the first 4 weeks of treatment were included in the safety analysis. The National Cancer Institute Common Terminology Criteria for Adverse Events (Version 4.0) were used to grade adverse events. All adverse events occurring during the first 4 weeks of treatment were included in the safety analysis. Hematological assessments, urinalysis, 12-lead electrocardiography, assessments of KPS performance status, and quality-of-life questionnaire administration were performed at the initial screening, on day 1, day 16, and at the end of the 28-day treatment.

### Statistical analysis

The response analysis was performed in patients whose tumor progression was assessed at at least one cycle of treatment (per protocol set). Categorical data are expressed as counts and percentages, and numerical data are presented as means with standard deviations or medians with interquartile ranges. The Kaplan-Meier method was used to calculate the median OS. The data cutoff date was February 11, 2022. All analyses were performed in SAR software (version 9.4).

## Results

### Patient characteristics

**Figure 1:**
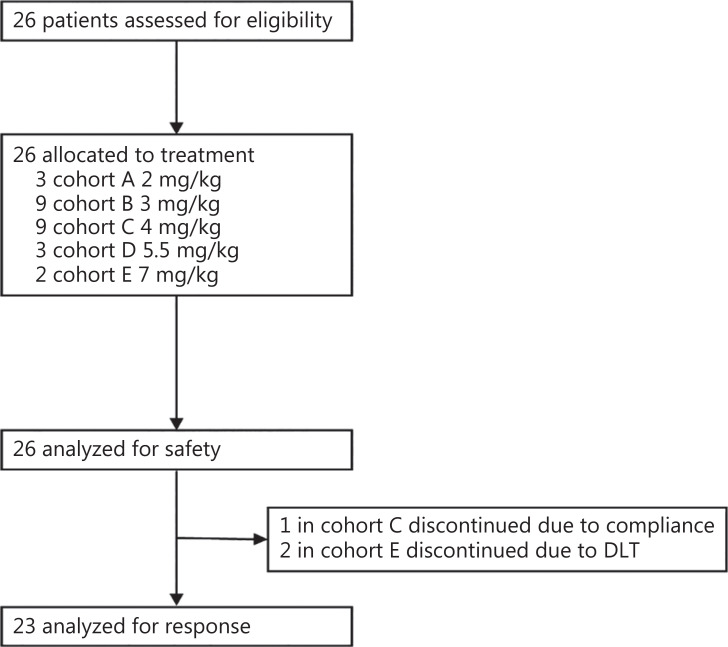
Randomization and treatment. The safety population included all patients who received at least one dose of chlorogenic acid. The population analyzed for response comprised all patients who received at least one cycle of chlorogenic acid and underwent tumor imaging (MRI).

Between April 2016 and June 2018, 26 patients with recurrent high-grade glioma after standard chemotherapy or radiotherapy were enrolled in the study and received intramuscular CGA injection (**Figure 1** and **Table 1**). Six of the 26 patients (23%) were histologically diagnosed with grade 3 glioma (**Supplementary Table S1**), and 20 patients (77%) were histologically confirmed to have grade 4 glioma. All patients received at least one chemotherapy or radiotherapy treatment before study enrollment. Of these patients, every third patient was sequentially enrolled in each dose cohort from 2 mg/kg to 5.5 mg/kg, and only 2 patients were enrolled in the 7 mg/kg cohort. The 3 mg/kg and 4 mg/kg cohorts were finally expanded to a total of 9 patients each.

**Table 1:** Demographic and baseline characteristics of the participating patients*

Characteristics	2 mg/kg (*n* = 3)	3 mg/kg (*n* = 9)	4 mg/kg (*n* = 9)	5.5 mg/kg (*n* = 3)	7 mg/kg (*n* = 2)
Gender					
Male	2	4	4	1	1
Female	1	5	5	2	1
Age, years					
Median	37	44	48	54	58
Range	(29–47)	(24–65)	(30–65)	(48–61)	(55–60)
KPS performance status, No. (%)					
<60	2 (67)	4 (44)	6 (67)	2 (67)	1 (50)
≥60	1 (33)	5 (56)	3 (33)	1 (33)	1 (50)
Histologic type, No. (%)					
Grade 3	0	3 (33)	2 (22)	1 (25)	0
Grade 4	3 (100)	6 (67)	7 (78)	2 (75)	2 (100)
Previous chemotherapy or radiotherapy treatments, No. (%)					
0–10	0	2 (22)	4 (44)	3 (100)	2 (100)
≥10, <20	0	4 (44)	4 (44)	0	0
≥20	3 (100)	3 (34)	1 (12)	0	0

*All patients were Chinese.

### Safety

Safety assessments involved analysis of all 26 patients. Twenty-three patients (88%) completed the study, and 3 patients discontinued treatment because of adverse events (2 patients) or compliance-related issues (1 patient). Among all 26 patients, 9 patients initially received injections at dose levels of 2, 3, and 4 mg/kg (3 patients for each dose level). Induration or pain at the injection sites was observed during the study, but the adverse events were low grade (grade 1 and 2) and well tolerated. The cohorts receiving 3 and 4 mg/kg were then expanded to include 9 patients in each cohort. Similar injection-related events were reported and were again well tolerated by the patients. However, treatment was discontinued in one patient because of poor compliance After completion of safety assessments, the doses were then increased to 5.5 mg/kg and 7 mg/kg. Two DLT events were observed in the 7 mg/kg cohort, in which grade 3 muscle-related injection induration was noted. The events resolved naturally without intervention after discontinuation of the test drug. Overall, the MTD was determined to be 5.5 mg/kg once daily.

Adverse events and treatment-emergent adverse events are summarized in **Table 2**. The most common adverse events were induration (92% of patients) and pain (12%) at the injection sites, swelling (4%), back pain (4%), insomnia (4%), hypertonia (4%), and increased blood creatinine phosphokinase levels (4%). Most of the adverse events were mild. Grade 3 induration at the injection sites was observed in 2 patients. No grade 4 adverse events were noted, and no patients discontinued from the study because of adverse events.

**Table 2:** Treatment-emergent adverse events*

Event	2 mg/kg *n* = 3, (%)	3 mg/kg *n* = 9, (%)	4 mg/kg *n* = 9, (%)	5.5 mg/kg *n* = 3, (%)	7 mg/kg *n* = 2, (%)	Total *n* = 26, (%)
Induration at injection site						
Any grade	1 (33)	9 (100)	9 (100)	3 (100)	2 (100)	24 (92)
Grade 3–4	0	4 (44)	3 (33)	1 (33)	2 (100)	10 (38)
Pain at injection site						
Any grade	0	3 (33)	0	0	0	3 (12)
Grade 3–4	0	0	0	0	0	0
Back pain						
Any grade	0	1 (11)	0	0	0	1 (4)
Grade 3–4	0	0	0	0	0	0
Swelling						
Any grade	0	1 (11)	0	0	0	1 (4)
Grade 3–4	0	0	0	0	0	0
Insomnia						
Any grade	0	0	0	0	1 (50)	1 (4)
Grade 3–4	0	0	0	0	0	0
Hypertonia						
Any grade	0	0	1 (11)	0	0	1 (4)
Grade 3–4	0	0	0	0	0	0
Elevated blood creatinine phosphokinase						
Any grade	1 (33)	0	0	0	0	1 (4)
Grade 3–4	0	0	0	0	0	0

*Calculations were based on 26 patients who received at least one dose of chlorogenic acid. Events were considered treatment-related, on the basis of assessment by the site investigators. The grade of adverse events was defined according to The National Cancer Institute Common Terminology Criteria for Adverse Events (Version 4.0).

### Pharmacokinetics

The pharmacokinetic parameters of the study drug are listed in **Table 3-1** and **3-2**. The plasma CGA concentration rapidly reached a peak level 0.5–1.5 h after a single intramuscular injection dose. Multiple doses of injection slightly decreased the T_max_ values. The maximum plasma concentration (C_max_) and exposure of CGA [area under the curve (AUC)] increased with dose elevation after single and multiple injections. No accumulation of the study drug was observed after multiple drug injections. CGA was rapidly eliminated from the plasma, with a t_1/2_ of 0.95–1.27 h on day 1 and 1.19–1.39 h on day 30 (**Table 3-1** and **3-2**), and no CGA concentration was detectable on days 9, 11, 13, 23, 25, 27, and 29 before CGA administration (**Table 3-2**). The pharmacokinetic parameters in the 7 mg/kg cohort on day 30 were not analyzed because of the discontinuation of treatment doses.

**Table 3-1:** Pharmacokinetic parameters of chlorogenic acid injection

Parameter	2 mg/kg (*n* = 3)	3 mg/kg (*n* = 9)	4 mg/kg (*n* = 8)	5.5 mg/kg (*n* = 3)	7 mg/kg (*n* = 2)
C_max_ (ng/mL)					
D1 (SD)	1300 (131)	2961 (835)	3244 (550)	3953 (796)	5495 (106)
D30 (SD)	1477 (420)	3313 (632)	3726 (822)	7653 (2811)	-
AUC_0-t_ (h*ng/mL)					
D1 (SD)	3202 (721)	7415 (1368)	8672 (1503)	10327 (1379)	14797 (1650)
D30 (SD)	3332 (518)	7334 (1839)	8351 (1304)	9968 (526)	-
AUC_0-∞_ (h*ng/mL)					
D1 (SD)	3343 (817)	7814 (1378)	8990 (1671)	10866 (1501)	15365 (2013)
D30 (SD)	3528 (519)	7707 (1983)	8844 (1618)	10414 (286)	-
T_max_ (h)					
D1 (range)	0.75 (0.50–1)	1.00 (0.50–1.50)	1.00 (0.75–1.50)	1.00 (1.00–1.50)	1.00
D30 (range)	0.33 (0.33–33)	0.50 (0.33–1.50)	0.50 (0.33–1.00)	0.17 (0.17–0.33)	-
t_1/2_ (h)					
D1 (SD)	1.12 (0.17)	1.27 (0.29)	1.02 (0.16)	1.27 (0.14)	1.11 (0.19)
D30 (SD)	1.19 (0.10)	1.34 (0.29)	1.36 (0.31)	1.39 (0.48)	-

-, not available; SD, standard deviation.

**Table 3-2:** PK analysis of CGA on different days of the first cycle

Dose cohort	Blood concentration before administration								AUC_0-last_		AUC_0-last_ratio (d30/d1)
	d9	d11	d13	d23	d25	d27	d29	d30	d30	d1	
2 mg/kg	/	/	/	/	/	/	/	/	3332.05	3201.98	1.04
3 mg/kg	/	/	/	/	/	/	/	/	7334.01	7414.69	0.99
4 mg/kg	/	/	/	/	/	/	/	/	8350.65	8672.05	0.96
5.5 mg/kg	/	/	/	/	/	/	/	/	9967.90	10327.33	0.97

d, day; AUC, area under curve.

### Patient response

**Figure 2:**
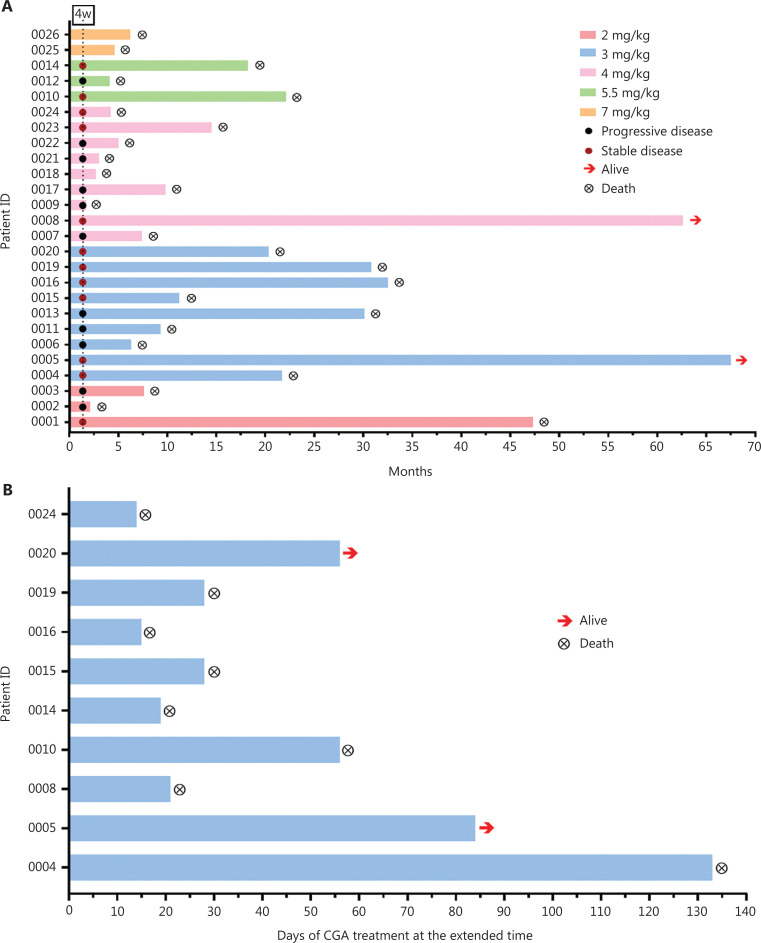
CGA treatment duration and patient response. (A) Summary of patient dose cohorts, patient response status assessed at the end of the first CGA treatment cycle, and the survival status after CGA treatment or combination treatment with standard-of-care therapeutics. Arrows indicate patients remaining alive at the most recent follow-up (February 11, 2022). Y axis, patient ID. X axis, time (month) from the start of CGA treatment until death. (B) Ten patients received CGA treatment at the extended time after the first cycle. Y axis, patient ID. X axis, days of CGA treatment at the extended time.

Twenty-three patients were analyzed for their response to CGA. The disease control rate after 4-week injections (the first cycle) was 52.1% (SD, 12) (**Figure 2A**). Of the 9 patients in the 3 mg/kg cohort, the brain tumor lesions in 3 patients shrank but did not reach the criteria for partial response at the end of first treatment cycle. Six patients (6 out 9, 67%) achieved SD in this cohort (**Table 4**).

**Table 4:** Summary of 17 patients with grade 4 glioma, including response assessment and overall survival, in the dose cohort, and after the first cycle and extended monotherapy with CGA

Patient ID	Dose cohort (mg/kg)	Primary diagnosis/pathology	Grade	Gender	Dosing C1D1	First treatment cycle (days)	Assessment after first cycle	Extended CGA only	Death date	Days (from start of CGA to death)^*^
**0001**	2	GBM	4	Male	2016/4/12	28	SD	No	2020/2/29	1419
**0002**	2	GBM	4	Male	2016/4/20	28	PD	No	2016/6/22	64
**0003**	2	GBM	4	Female	2016/6/7	28	PD	No	2017/1/22	230
**0005**	3	GBM, containing oligodendroglioma	4	Female	2016/7/28	28	SD	Yes	NA	NA
**0006**	3	GBM	4	Male	2016/8/11	28	PD	No	2017/2/15	189
**0007**	4	Astrocytoma mixed with GBM	4	Male	2016/9/23	28	PD	No	2017/5/2	222
**0009**	4	GBM	4	Female	2016/12/22	28	PD	No	2017/2/11	52
**0010**	5.5	GBM	4	Female	2017/2/10	27	PD	Yes	2018/12/5	664
**0012**	5.5	Astrocytoma mixed with GBM	4	Male	2017/2/24	28	PD	No	2017/6/28	125
**0013**	3	Anaplastic astrocytoma mixed with GBM (right frontal)	4	Female	2017/3/9	28	PD	No	2019/8/29	904
**0019**	3	GBM with massive necrosis	4	Female	2017/6/6	28	SD	Yes	2019/12/17	925
**0021**	4	GBM (right frontal)	4	Male	2017/6/22	28	PD	No	2017/9/21	92
**0022**	4	GBM (right frontal)	4	Female	2017/7/6	28	PD	No	2017/12/3	151
**0023**	4	GBM (right thalamus)	4	Female	2017/7/7	28	SD	No	2018/9/15	436
**0024**	4	GBM	4	Female	2017/7/10	28	SD	Yes	2017/11/13	127
**0004**	3	Astrocytoma with mucus-like transformation	4	Male	2016/7/27	28	SD	Yes	2018/5/10	653
**0015**	3	Anaplastic astrocytoma with focal necrosis	4	Female	2017/3/24	28	SD	Yes	2018/2/24	338

C1D1, cycle 1 day 1; NA, not applicable. ^*^Including CGA monotherapy, and its combination with TMZ and other standard-of-care therapeutics after the first treatment cycle of CGA until the follow-up on February 11, 2022.

**Figure 3:**
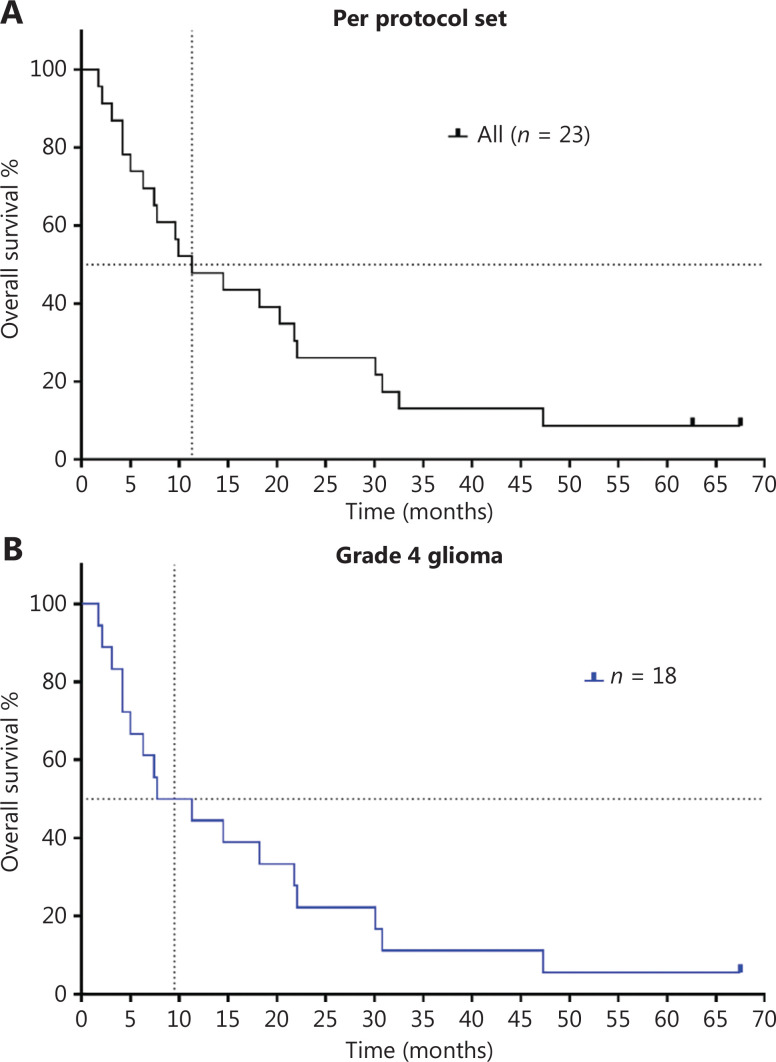
Kaplan-Meier curves of overall survival in patients with recurrent high-grade glioma who were treated with chlorogenic acid. (A) OS of per protocol set population (*n* = 23); (B) OS of patients with grade 4 glioma, who received at least the first cycle of CGA. These patients included those with glioblastoma and astrocytoma (*n* = 18).

According to the protocol and physician’s judgement, after 4-week treatment, the patients could continue to receive the CGA alone or in combination with other treatments. All the patients were followed up until their death. Among these patients, 10 received extended CGA monotherapy for a median of 28 days (range 14–133 days) after the first cycle (**Figure 2B**). The 1-year survival rate of these patients in follow-up was 47.8%, and the 2-year survival rate was 26.1%. After a 5-year follow-up, 2 patients were alive (1 patient with grade 3 glioma, ID 0008 and 1 patient with grade 4 glioma, ID 0005). The estimated median OS was 11.3 months for all patients (**Figure 3**). Of the 18 patients with grade 4 glioma, the median OS was 9.5 months.

**Figure 4:**
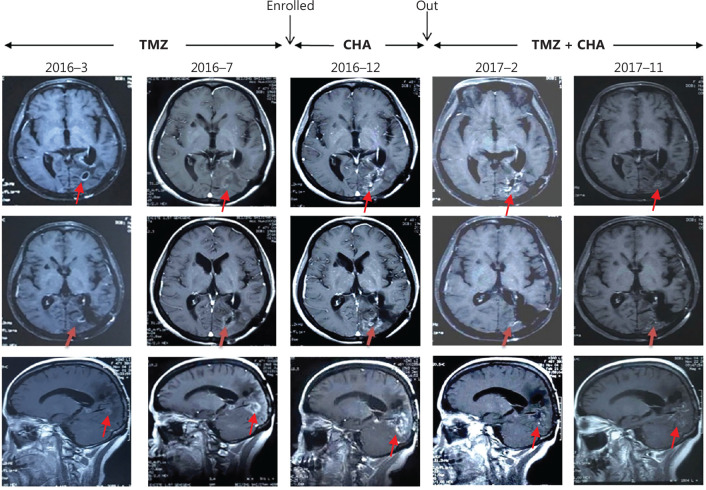
MRI images (arrows indicate tumors) obtained at baseline, 4 weeks of treatment, and follow-up of a representative patient (ID 0005) ww chlorogenic acid treatment. Red arrows indicate tumors.

Interestingly, the patient with glioblastoma (GBM) (patient ID 0005) was resistant to TMZ before enrollment. After CGA treatment for 4 weeks, the tumor lesions remained stable. Subsequently, this patient continued to receive CGA treatment in combination with TMZ, and the tumor lesion began to shrink (**Figure 4**). After 18 months of combination treatment, MRI indicated that a new lesion had appeared in the posterior temporal lobe, and the patient started metformin treatment in combination with TMZ and CGA. The dose of metformin was 0.25mg twice per day. In July 2019, MRI indicated that the lesion was stable, and the patient continued to receive triple combination treatment until the tumor progressed (the last MRI was conducted in October 2020) (**Supplementary Figure S1**).

## Discussion

Patients with recurrent high-grade glioma (grade 4 glioma is the most common) after standard chemotherapy or radiotherapy generally had a median OS of 5.7 to 7.5 months^4,12,13^. Beyond standard chemotherapy and radiotherapy, no other standard therapy is currently available for treating this deadly disease. CGA is a natural product that is widely found in edible plants. Preclinical studies have demonstrated that CGA exerts antitumor activity, possibly because of its ability to inhibit tyrosine kinases, matrix metalloproteinases, HIF-1α, and AKT, and more importantly to repolarize macrophages and enhance anti-cancer immunity in glioma^6,8,9,14,15^. These findings provided a rationale for investigating CGA as a potential novel therapy for treating recurrent glioma. However, preparing CGA from plants in large quantities is challenging, and the extracts often have low water solubility and can cause respiratory allergy, thereby limiting their use in clinical settings. The injectable form of CGA used in this study was prepared with high-quality CGA (with purity greater than 99.9% and an absence of allergenic substances, after the improvement of industrial scale manufacturing and purification procedures). Allergy testing in our preclinical study demonstrated that CGA with a purity greater than 99.9% did not induce systematic allergy. Thus, this injectable form of CGA is the first of this CGA class of compounds to be tested in clinical trials evaluating anti-tumor activity. To evaluate its safety in patients, we also performed allergy tests for CGA to ensure that no allergy occurred in the screened patients, in agreement with the preclinical results.

To our knowledge, this article also provides the first report of CGA therapy in patients with recurrent high-grade (grade 3 and 4) glioma in a clinical trial setting. In this phase I trial, CGA was well tolerated and showed encouraging antitumor activity in patients with recurrent glioma. The MTD for CGA injection was determined to be 5.5 mg/kg. The most common drug-related adverse events were injection-related reactions (i.e., induration: 92%; pain: 12%). No grade 3 or 4 adverse events were observed for other adverse events except induration at the injection sites. The occurrence of these injection-related events correlated with the volume of the injected substance. Most adverse events were well tolerated by the patients, who recovered without medicinal intervention. Pharmacokinetic analysis indicated a linear pharmacokinetic profile with no accumulation after multiple injections; no CGA was detectable on days 9, 11, 13, 23, 25, 27, and 29 before CGA administration.

In a preclinical *in vivo* animal study, the effective dose of CGA was found to be between 20 and 40 mg/kg. On the basis of human equivalent dose conversion, the efficacious dose in humans was estimated to be 2–4 mg/kg. In the expansion cohort, 9 patients were selected for PK, safety studies, and preliminary efficacy evaluation to provide information to support a phase 2 study. Thus, 5.5 mg/kg is not a strictly defined MTD but is limited by injection site related adverse events. In future related trials, injections at multiple sites may be tested to determine whether the treatment dose could be increased and, if so, whether patients might achieve any benefits with doses greater than 5.5 mg/kg.

Preclinical studies have shown that the T1/2 of CGA in animals is 40–50 minutes; and the T1/2 in this human clinical trial was approximately 1 h. Chlorogenic acid was rapidly eliminated *in vivo*. However, because CGA’s mechanism involves activation of immune and metabolic pathways that exert anti-tumor roles, once-daily administration can maintain an activated immune state in the body and consequently may help address issues with rapid elimination of CGA.

In our preclinical study, the main metabolic pathway of CGA *in vivo* was found to be the O-methylated metabolic pathway, whose products are 3-O-ferulic quinic acid and 4-O-isoferulic quinic acid. The above metabolites were also detected in urine samples analyzed in this clinical trial. Thus, we speculate that similar pathways might be involved in CGA metabolism in patients.

Although the size of this trial was small, the study did indicate that 52.1% of patients (12 of 23) achieved SD at the end of the first cycle of treatment. Importantly, the estimated median OS was 11.3 months for all patients and was 9.4 months for the 18 patients with grade 4 glioma. Furthermore, the 1-year survival rate was 47.8%, and 2 patients were alive at the cutoff day of February 11, 2022. With respect to the median OS (5.7 to 7.5 months) for patients with recurrent high-grade glioma treated with standard-of-care therapeutics, the median OS was prolonged after CGA treatment. These preliminary results are very encouraging and may aid in providing necessary care for a deadly malignancy.

The localized reactions at the injection site might possibly have been due to 2 factors: (1) CGA (injection) is mildly acidic and may elicit local inflammatory reactions in the skin and muscle at the injection site, and (2) CGA is a small molecule immunomodulator. In addition to being absorbed into the blood, a small amount of the drug remaining in muscles may trigger a localized immune response at the injection site. In this clinical trial, a topical hot compress was used to facilitate drug absorption and decrease the local inflammatory response to mitigate the frequency and extent of these adverse events.

No long-term adverse effects of CGA were observed in the long-term follow-up of the participants in this trial. The clinical observations were consistent with the preclinical animal safety evaluation of CGA, in which no apparent toxic adverse effects were found in long-term toxicity studies in rats (180 days) and beagle dogs (270 days), on the basis of analysis of hematological parameters and pathological tissue assessment at the end of treatment, thus indicating that CGA was well-tolerated.

Notably, after the first 4 weeks (the first cycle) of CGA monotherapy, per protocol and physicians’ judgement, patients were eligible for treatment with CGA in combination with the standard-of-care therapeutic TMZ, because of the features of this advanced deadly disease. The combination of CGA and TMZ helped prolong OS. Patient 0005, who had survived as of the cutoff day, benefited from a treatment regimen moving from CGA monotherapy to CGA and TMZ in combination, and finally to triple combination therapy with metformin. This case study sheds light on the potential benefits of combining CGA with standard chemotherapy for glioma treatment. The underlying mechanism is currently under investigation.

Our study has several limitations. This was a phase I study, and the sample size was too small to accurately assess the OS and progression-free survival of patients with recurrent high-grade glioma. Similarly, the protocol design allowed for combination treatment with CGA after the first cycle, thus complicating the assessment of efficacy or OS associated with CGA monotherapy. Beyond CGA, in the follow-up period, the combination of TMZ with PCV or bevacizumab could also be applied. Such combinations are recommended in clinical diagnosis and treatment guidelines.

On the basis of our follow-up observations, patients who continued CGA monotherapy or combination treatment with standard-of-care therapy after the first cycle appeared to have good responses. This clinical observation is consistent with those from previous publications, in which patients with recurrent glioma have been found to respond to TMZ rechallenge^16^. A future study may elucidate the mechanism of action of the combination of CGA with TMZ, or sequential treatments with these 2 mechanistically distinct agents.

During the trial period, 229 patients receiving supportive therapy beyond CGA or standard-of-care regimens, such as TMZ, were documented. The supportive medicines included steroid hormone drugs and recombinant human granulocyte colony stimulating factor (GCSF). Steroid hormone drugs were immunosuppressive, and were used in 21 cases during the trial, accounting for 9.1% of all patients receiving supportive therapies. However, the steroid treatment was short term, for 3–5 days, and was used primarily to control the symptoms of cerebral edema and intracranial hypertension; the drugs were discontinued after the symptoms had been controlled.

In contrast, recombinant human GCSF is an immune-enhancing agent, which was applied in 6 cases and accounted for 2.6% of all patients receiving supportive therapies. GCSF was also administered short term, for 1–3 days, to balance granulocyte levels in patients with advanced tumors. Given that these immune-modulatory agents were used only as needed and for very short periods of time during the trial, their possible effects on CGA function cannot be excluded; however, such effects are expected to be minimal because of the reasons stated above.

## Conclusions

In conclusion, this dose-escalation phase I study demonstrated that the injectable form of CGA was well tolerated in patients with recurrent high-grade glioma. Preliminary anti-tumor activities, and potentially OS benefits for these heavily pre-treated patients, were observed. Deeper responses to CGA monotherapy may be investigated and confirmed in a phase 2 clinical trial.
