# Supplementary material for: Phase I study of chlorogenic acid injection for recurrent high-grade glioma with long-term follow-up
Source: Cancer Biol Med. 2023 Jun 22;20(6):465–76. doi: 10.20892/j.issn.2095-3941.2022.0762 (PMC10291982; doi:10.20892/j.issn.2095-3941.2022.0762)
Supplement: Supplementary file 1 [file cbm-20-465-s001.pdf]

Supplementary materials

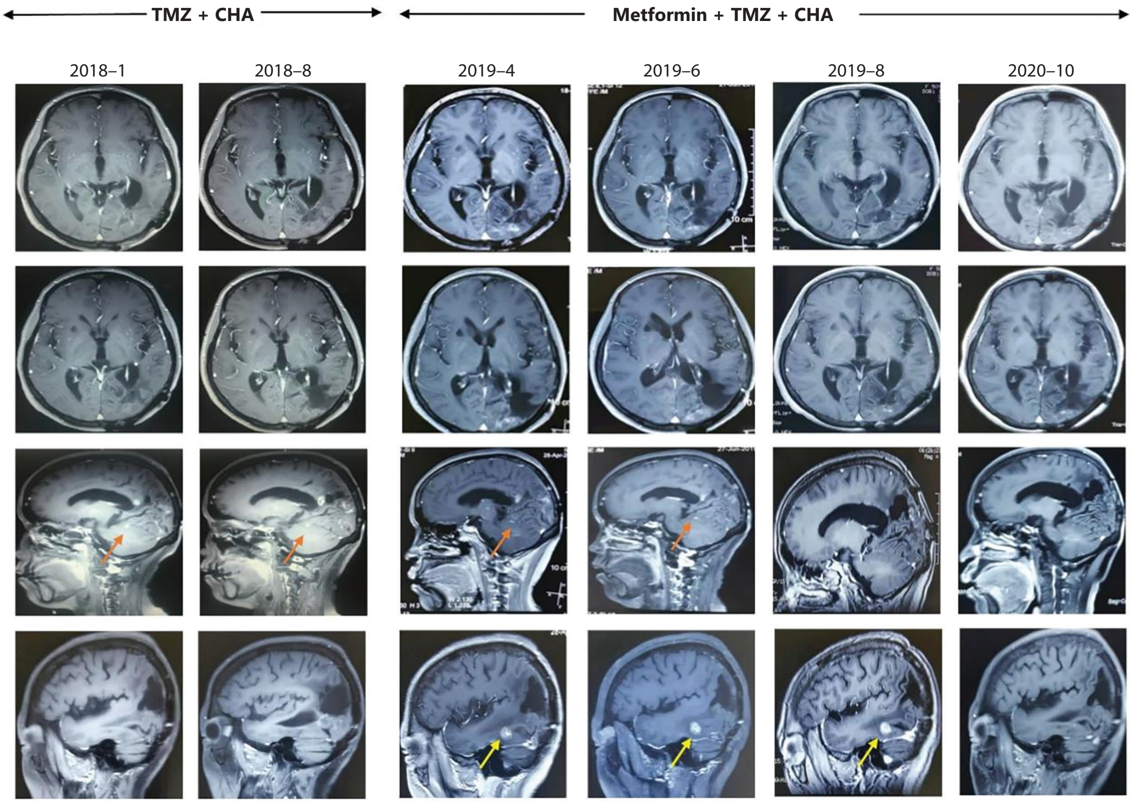

Figure S1 MRI images (arrows indicate tumors) of 4-year follow-up in a representative patient who received chlorogenic acid treatment. In January 2018, MRI indicated a new lesion in the cerebellum (yellow arrow). In April 2019, MRI showed a new lesion in the posterior temporal lobe, and the patient started to take metformin (0.25 mg twice per day) in combination with TMZ and CGA In 2019, MRI indicated that the lesion was stable, and the triple combination treatment was continued.

Table S1 Summary of 6 patients with grade 3 glioma, including response assessment and overall survival, in the dose cohort, and after the first cycle and extended monotherapy with CGA

Patient ID	Dose cohort (mg/kg)	First diagnosis/pathology	Grade	Gender	Dosing C1D1	Treatment days (first cycle)	Assessment after first cycle	Extended CGA only	Death date	Days (from start of CGA to death)*
0008	4	Oligodendrocytes, local growth activity	3	Male	2016/12/21	28	SD	Yes	NA	NA
0011	3	Bifrontal lobe and callosum anaplastic astrocytoma, tumor cells diffuse proliferative infiltration and accumulation of brain surface	3	Male	2017/2/11	28	PD	No	2017/11/24	287
0014	5.5	Astrocytoma (right frontal lobe)	3	Female	2017/3/16	28	SD	Yes	2018/9/13	547
0016	3	Oligoastrocytomas, localized cell density increase	3	Female	2017/5/17	28	SD	Yes	2020/1/16	975
0017	4	Anaplastic astrocytoma	3	Female	2017/5/19	28	PD	No	2018/3/10	296
0020	3	Anaplastic astrocytoma	3	Male	2017/6/8	28	SD	Yes	2019/2/6	609

NA, not applicable. *Including CGA monotherapy, and its combination with TMZ and other standard-of-care therapeutics after the first treatment cycle of CGA until the follow-up on February 11, 2022.
